# Returning individual‐specific results of a dementia prevalence study: insights from prospective participants living in Switzerland

**DOI:** 10.1002/gps.5416

**Published:** 2020-09-11

**Authors:** Marta Fadda, Maddalena Fiordelli, Rebecca Amati, Ilaria Falvo, Aliaa Ibnidris, Samia Hurst, Emiliano Albanese

**Affiliations:** ^1^ Institute of Public Health, Faculty of Biomedical Sciences Università della Svizzera italiana Lugano Switzerland; ^2^ Institute of Communication and Health, Faculty of Communication Sciences Università della Svizzera italiana Lugano Switzerland; ^3^ Institute for Ethics, History and the Humanities University of Geneva Geneva Switzerland

**Keywords:** dementia, epidemiology, individual‐specific results, older adults, qualitative research, Switzerland

## Abstract

**Objectives:**

To explore prospective participants' preferences regarding the return of their individual‐specific results from a dementia prevalence study (a probabilistic diagnosis of dementia).

**Methods/Design:**

We conducted a qualitative study with 22 individuals aged 45 to 86 and resident in the Canton of Ticino (Switzerland). Participants had previously joined the validation phase of an epidemiological study into dementia and its impact.

**Results:**

We found that individuals welcome the return of their individual‐specific results, provided these meet a number of validity, clinical, and personal utility criteria. They justify researchers' duty to return study findings with the principles of beneficence (eg, providing information that can help participants' medical decision‐making) and justice (eg, acknowledging participants' efforts to help research by sharing their personal information). Furthermore, individuals anticipate societal benefits of the return of individual specific study findings, including improved interpersonal relationships among individuals and decreased dementia‐related stigma.

**Conclusions:**

Our findings suggest that researchers should address the return of individual‐specific study results early on during study design and involve prospective participants in identifying both the conditions under which results should be offered and the perceived individual and societal benefits returning can have.

**Key points**

- Dementia research and innovation is action area 7 of the WHO Global action plan on the public health response to dementia
- Participants of dementia studies may have expectations to receive personal gain from participation, such as their individual‐specific research results
- Principal investigators (PIs) of dementia studies should offer return of individual‐specific study results after identifying, together with prospective participants, the conditions under which results should be offered

## 1 INTRODUCTION

Dementia research and innovation correspond to the seventh action area of the World Health Organization (WHO) Global action plan on the public health response to dementia.^1^ Advancing research is considered an urgent and crucial matter, in order to not only decrease dementia incidence but also improve the lives of patients, their families, and caregivers.^1^, ^2^ Epidemiological research is one of the plan's priorities.^3^, ^4^ Epidemiological data can provide information on the prevalence and incidence of the disease but also its impact, including the direct and indirect costs.

Effective progress in this area also depends on the promotion of participation in research. In fact, the well‐known obsolescence of dementia‐related epidemiological data in Europe^5^ is due to a decrease in participation rates in epidemiological studies over the past 30 years, which has witnessed an even steeper decline in more recent years.^6^, ^7^ The success of population studies depends on the voluntary participation of individuals who donate their time and their personal health information, often accepting a certain degree of risks to their welfare. Therefore, researchers should take into account legitimate expectations of individuals to receive some personal gain from participation, such as research results or information on their health.^8^ Evidence suggests that current dementia research ethics policies and norms are not aligned with participants' preferences and may hinder equitable opportunities to take part in epidemiological research.^9^, ^10^

Among the reasons to participate in DNA biobank studies, expectations of personal benefit through health information are prominent.^11^, ^12^, ^13^ Willingness to participate is higher when participants are offered a chance to know their individual‐specific results. Consensus statements indicate that the return of research results should occur when the findings are clinically relevant.^14^, ^15^, ^16^, ^17^ Recently, scholars have highlighted the urgent need for guidance in related decision‐making contexts, such as when to provide family members with access to health‐related data of dementia patients and how to manage the return of individual results from dementia research.^18^ Lack of clarity on how to meaningfully interpret positive results in a clinical sense, coupled with fears of causing anxiety or depression to subjects have so far prevented most investigators from disclosing individual‐specific research results to dementia research participants.^19^, ^20^, ^21^, ^22^ However, little has been done to address such issues in dementia epidemiological research. We conducted a qualitative study to explore preferences regarding and understanding of the return of individual‐specific results, in view of an epidemiological study into dementia and its impact, whose primary outcome is prevalence of dementia based on a probabilistic dementia diagnosis.

## 2 METHODS

### 2.1 Study design

This article presents a follow‐up investigation of a validation study conducted between March and October 2019 with 160 dyads in the Canton of Ticino, Switzerland. Each dyad consisted of an old person (aged +65 years) and his/her informant (ie, a carer, spouse, or child). Therefore, in the original study, the number of informants was equal to the number of older adults (n = 80 in each group). In the present investigation, we conducted semi‐structured, in‐depth interviews with a sample of the validation study's participants. We included both participants and their informants to generate a more comprehensive understanding of their attitudes, thoughts, and preferences regarding the return of individual‐specific results.^23^

### 2.2 Study participants

Inclusion criteria for participants were age (being 65 or older) and place of residence (Ticino). Informants had to be older than 18 years. In the informed consent for the validation study, participants gave permission to be contacted for a follow‐up interview. No individual‐specific results were offered in the validation study. Of the 320 participants of the validation study (160 dyads), 35 individuals provided their contact details and were contacted by a member of the research team (RA), who provided a description of the present study, and reference to its ethical safeguards. We offered no financial incentives for participation. Recruitment lasted between December 2019 and January 2020.

### 2.3 Data collection

The interviews were conducted in Italian and face‐to‐face by a member of the RA between December 2019 and January 2020, either at the participant's home or the University, according to participants' preferences. Three couples requested to be interviewed together. In these cases, both participants took turn to answer to all questions posed by the interviewer, resulting in two answers for each question, which were analyzed independently. The interview guide was designed to foster an in‐depth conversation and consisted of preset open‐ended questions formulated to elicit their preferences on *what*, *how*, to *whom*, and *when* research results (both general and individual‐specific) should be returned. A second set of open‐ended questions explored participants' perspectives on *why* results should be returned. These include personal views and interpretation of the nature and value of the return of the results, personal justification in favor or against the return, and anticipated feelings (see Appendix 1 for the interview grid). Participants were asked to imagine that they might be receiving information on whether they are likely to have dementia or not. We followed Holstein and Gubrium's “active interview” model, which conceptualizes the interviewer and interviewee as equal, collaborative partners in the social production of meaning around a given research topic.^24^

After permission from participants, we digitally video‐recorded all interviews. One member of the RA transcribed them verbatim, while a second member (IF) cross‐checked the recorded interviews to guarantee accurate documentation of the discussion. To determine the point of data saturation, we relied on the concept based on thematic redundancy, or inductive thematic saturation.^25^, ^26^ In this model, which relates to the emergence of new codes or themes, saturation is confined to the level of analysis and data collection is interrupted when no novel insights can be extracted from the data.^26^ We reached saturation of the data after the 11th interview. We collected data on participants' sociodemographic characteristics at the end of each interview.

### 2.4 Data Analysis

Two members of the RA (MF* and IF) independently performed an inductive thematic analysis of the 19 transcripts. We followed the six‐stage comprehensive thematic analysis approach developed by Braun and Clarke.^27^ The key phases of the coding process include familiarizing with the content of the transcripts, highlighting meaningful quotes regardless of their length, condensing them under a number of labels, organizing the generated labels hierarchically, creating relationships between them, and identifying remarkable quotations to represent thematic similarities, differences, and contradictions. This method allows to unveil themes that may not have been covered by established theory.^28^, ^29^ To validate the results, comparisons between the two coders took place multiple times in‐between each of the above‐mentioned phases, so that themes, labels, and quotations were constantly discussed, and interpretation discrepancies resolved through dialog and reference to the transcripts. We performed the analysis with the qualitative research software NVivo.^30^ No translation of the transcripts was needed, as the analysis was conducted in the original language. This article follows the Standards for Reporting Qualitative Research (SRQR).^31^

## 3 RESULTS

### 3.1 Participants' characteristics

The final sample included 22 participants (11 women), including three couples that requested to be interviewed together. The mean age was 71 years (SD = 9.3; range = 45‐86). Most participants had completed secondary school (n = 11), were retired (n = 17), and resident in urban districts (n = 13; see Table 1 for an overview of participants' sociodemographic characteristics).

**TABLE 1:** Characteristics of participants (N = 22)

	n (%)^a^
Gender	
Male	11 (50.0)
Female	11 (50.0)
Age	
45‐64	4 (18.2)
65‐69	6 (27.3)
70‐74	4 (18.2)
75‐79	4 (18.2)
80‐84	3 (13.6)
85‐86	1 (4.5)
Nationality	
European	4 (18.2)
Swiss	18 (81.8)
District of residence	
Bellinzona	4 (18.2)
Laventina	2 (9.1)
Locarno	2 (9.1)
Lugano	14 (63.6)
Highest level of education	
Primary	1 (4.5)
Secondary	11 (50.0)
University	9 (41.0)
Occupation	
Retired	17 (77.3)
Volunteer	5 (22.7)

^a^ Percentages may not total 100% due to missing values.

### 3.2 Preferences regarding the return

A clear difference between aggregated and individual results emerged from the interviews. All participants welcomed the return of the overall study findings, with most of them reporting that they would like to be informed also on intermediate results and not only final ones. Reasons included a desire to be regularly updated on the study, know better what they are contributing to and what investigators have discovered. A small minority expressed a preference for a public disclosure of the aggregate results rather than individual‐specific ones, which would allow them to receive peer support from other individuals who are experiencing the same situations and emotions.

The focus of this article will be on individual‐specific results. Most participants were in favor of the return of their individual‐specific results. The majority stated that they expected them, even though they favored different approaches to disclosure. Most (n = 12) reported that they would not be afraid of the disclosure and had a strong desire to know their personal results. Four participants also reported anticipated fear with respect to the disclosure but declared that they would nevertheless prefer to know the results in order to act. Finally, two participants reported they would be afraid and prefer not to know, stating they would not be able to psychologically manage a risk of dementia.

Preferences on how results should be returned varied across participants, and according to the type of results. Most participants stated that they would accept their results being communicated via e‐mail, postal letter, or phone call in case they were negative (no risk of dementia), but agreed that, in case of positive results, a face‐to‐face meeting with the investigator would be more appropriate.

With regards to whom they would like their individual‐specific results to be shared with, most mentioned their spouse and children. Only those reporting a positive relationship with their family doctor would share their results with him/her and a few reported that they would like themselves to be the only recipients of the results. In terms of timing, all reported that disclosure should happen as soon as possible if results were positive.

### 3.3 Understanding of the nature of the return

We categorize participants' understanding of the nature of the return of research findings around three broad themes: (a) a matter of transparency; (b) a matter of reciprocity; and (c) a matter of relationship.

### 3.4 A matter of transparency

The majority of participants reported that researchers have a moral duty to return individual‐specific results based on transparency towards study participants.

“[Return of study results] must be agreed in a very clear way. […] We should be grateful to those who warn us or make us aware of some problems, no? […] I find it very positive when someone, even a friend, makes us aware or points out to some deficiencies. I believe that this, if a person reasons in an objective way, is fair and it is something that must happen”. (P2, 80, male, university, older adult).

Some participants recognized that researchers should be transparent on results that may have clinical utility.

“If there were general indications… to understand if there is a beginning of an aging process that is not really healthy like Alzheimer's, like dementia…”. (P16, 67, female, secondary school, older adult). or, as the following caregiver reported, that may reduce uncertainty.

“[…] When you enter this problem, anything will give you joy, because it's just so hard to go on”. (P1, 73, male, secondary school, informant).

Two participants also noted that results could not only prompt him/her to seek help but also have an important impact on reducing the stigma and isolation associated with dementia and improve relationships with friends and family members.

“Well, it helps… that is, if I know what I have, what is happening, I can ask for help where they can give it to me, I can talk about it with my friends, they will understand me better, I will no longer see those looks like <<What is it? What are you doing?>> It will be a much more open and sincere relationship with others, rather than denying, having to hide and not wanting to accept… I think I should invest much more energy on talking about it openly rather than in hiding and camouflaging everything, so that I can channel my energy to help me deal with the situation”. (P6, 69, female, university, older adult).

### 3.5 A matter of reciprocity

Most participants reported that researchers have a moral duty to return study results because this satisfies a principle of reciprocity or mutual exchange. Participants share something with researchers and should, therefore, receive something back. Moreover, failing reciprocity would hinder participation in future, similar studies.

“[…] someone gives you [something]… A company gives you a small amount, [you should say] at least a ‘thank you’. If you do not do it, you lose. Therefore, there needs to be this exchange. If you give me 20 francs for my association, I at least must thank you. Thank you for being close to our association. But if I do not do anything, next time you will say <<what do these guys want again?>>”. (P1, 73, male, secondary school, informant).

In addition, a restricted number of participants reported that the principle of reciprocity is not absolute but should be balanced against the validity and the actionability of the results. They reported to be aware of the limitations of the test (in terms of diagnostic uncertainty) which would require a follow‐up with further examinations.

“I think it is a matter of *do ut des*. I mean, we as participants have consecrated time and commitment to answer these questionnaires. Even if this scares us off, we surely also expect a non‐binding answer, with all the necessary precautions, possibly with a recommendation to see one's family doctor or discuss the results that have emerged… therefore, my opinion would be that it would be an act of courtesy from your side” (P2, 80, male, university, older adult).

Despite recognizing the duty to return study results, one participant noted that the scope of a study is to generalize knowledge and not to identify deficits in single individuals. According to this participant, offering options to communicate results should not be framed as an incentive to participation, as this risks to represent a source of exploitation of those participants who would join the study only to know about their results.

“I have some doubts regarding the fact that you are the one piloting the participant, because participants decide to join your initiative in order to provide information and allow your study to have solid basis, they do not come to you to be helped basically, therefore I do not believe that you should assume a guiding role on that towards participants”. (P2, 80, male, university, older adult).

### 3.6 A matter of relationship

Almost all participants reported that, for optimal disclosure, it is central that researchers establish a personal relationship with participants early on during recruitment.

“Now I speak very personally: this experience has shown me that what really matters is contact with the person”. (P16, 67, female, secondary school, older adult).

For this participant, it is important that contact is not only personal, but also open and that meetings can be scheduled in a flexible fashion.

“If you would like to come, gladly, that would certainly make us glad… but perhaps your visit, after [doing] certain things, we can always meet, that is very nice, gladly. It's interesting so that it doesn't stay a hybrid thing. Exactly the contact with the other part who is working, that is nice. […] I am always open in any moments… As we did it today: ‘we have this thing here’, we meet… ‘we have these results here, we would be pleased to come’ and then we meet”. (P1, 73, male, secondary school, informant).

For the following participant, meetings with the RA should be nonjudgmental and should constitute an opportunity to express one's problems.

“It would be nice for those who are interested to personally meet, to express their problems”. (P13, 77, male, secondary, informant).

Half of the participants reported their desire to maintain a relationship with the study team also after the interview is concluded and the results are disclosed. Participants recognized that results can potentially reveal a risk of dementia which can be difficult to manage from a both behavioral and psychological point of view.

“Research [should invest] on individuals […] as politicians always do when they want to get votes, they are all there, and after that they disappear. You are abandoned”. (P1, 73, male, secondary school, informant).

For this reason, participants reported that they would like to receive the researchers' support, which they described in two main ways. About a quarter of participants expressed a desire to receive remote or face‐to‐face personal feedback on how their individual‐specific results are different compared to the average, and advice on whom to consult and how to mitigate the risk.

“If it hadn't gone so well it would be important to understand how to move in the future, what kind of measures, what kind of solutions I should take”. (P12, 74, female, university, older adult).

A restricted number of participants reported a preference for a face‐to‐face, empathic consultation.

“If I had to go back to what I experienced with that biopsy [performed not in the context of the present study], I would probably not do it again, but this is not due to the biopsy itself but rather to the lack of emotional support, and for me emotional support is fundamental. (P16, 67, female, secondary school, older adult).

## 4 DISCUSSION

The goal of this study was to explore participants' preferences regarding the return of their individual‐specific results in a prospective study on dementia prevalence. We found that the return of individual‐specific results should repose on two main principles, transparency and reciprocity, and be dialogic. Investigators should disclose anything that could inform health‐related decision‐making, and the benefits that may derive from individual uses of participants' information should be mutual. Next, participants reported that investigators should assess the validity, clinical utility, and actionability of study findings prior to offering their return. Finally, return should occur in the context of a personal, open, and supportive relationship with the investigator.

The idea of justifying the return of research findings with the arguments on transparency and reciprocity is not new to the literature. In the context of genomics, studies found that participants have several health‐related and personal reasons for wanting individual research results, and researchers recognize that the highest benefit is helping treat or prevent disease.^32^, ^33^, ^34^, ^35^, ^36^, ^37^ In the biobanking context, providing participants with their individual‐specific study results that are valid and with potential clinical utility, is perceived by both researchers and participants as a means of demonstrating respect and gratitude for their contributions.^38^, ^39^, ^40^ Studies have shown that provision of such information may lead to greater trust, accountability and engagement in research, which is, in turn, a strong predictor of research participation.^8^ Finally, the obligation to respect participants' ownership of the information they provided—and thus offer “return” rather than “disclosure”—is consistent with the shift towards participant engagement.^41^ Traditionally, epidemiologists strive to maximize the potential benefits of research by communicating results to study participants in a timely fashion.^42^ This study expands the current interpretation of the principle of beneficence in epidemiological research.^42^ Our results indicate that the return of study results can also contribute to reducing stigma, through improved awareness and understanding of dementia at a collective level. This is consistent with the pivotal role of dementia awareness of the WHO public health response to dementia.^1^ Our findings on the dialogic context that is necessary for the return of study results are in line with previous investigations on the implications of a dementia diagnosis and its communication with patients and family members. In the clinical context, studies highlighted the difficulties in the communication process with patients,^43^ the necessity to understand what the diagnosis means to them (including the impact of disclosure),^44^ and the value of peer support for people with dementia and their carers as a postdiagnostic intervention.^45^ Our findings suggest that, in the epidemiological context, return of study findings should occur within an optimal relationship with the investigator, one that does not fade after results are disclosed. If results suggest an increased risk of dementia, investigators should have a role in helping them to manage this risk by offering emotional support and medical advice.

People might frame data ownership as private property, thus perceiving that data belong first and foremost to themselves.^46^ This could explain the expectation that researchers strictly adhere to a principle of transparency. Furthermore, individuals contributing to research with their health‐related information often do so because they are curious about themselves.^11^, ^12^, ^13^ Frustration and loss may result when investigators take but do not share. This explains why participants argued for a principle of reciprocity. As in the clinical context, study participants are aware of the potential difficulties in understanding and managing dementia‐related results and the impact of their disclosure on different levels, including potentially on health costs.^44^, ^45^ In Switzerland, private health insurance is compulsory for all residents, and a diagnosis of dementia may lead to additional costs for individuals related to copay for clinical assessments and exams. However, dementia treatments and most care and nonpharmacological interventions are fully covered by health insurance after a clinical diagnosis made by a specialist. In addition, a dementia diagnosis has no direct impact or implications for fitness‐to‐drive assessments and license. Nonetheless, it is likely that participants may expect that interpersonal contact with the investigator is established early on and maintained throughout the entire study to discuss the implications of both negative (ie, no dementia) and positive results.^47^

Based on our results, implications are to be noted for both theory and practice. In terms of theory, our results enrich our understanding of the conditions under which disclosure of individual‐specific study results should occur. To respect the ethical principle of nonmaleficence, and thus prevent participants' exposure to unnecessary risks, investigators have an obligation to take into account the test validity, results' actionability and significance, and their personal and clinical utility.^48^ Our findings add that, when such criteria are taken into account, the interpersonal component of the disclosure should be integrated. In terms of practice, investigators should discuss the issue of the return of research findings early on during study design and address it with the study team. Since the return of individual‐specific research results necessarily requires the diversion of some resources from the primary goal of the research,^49^, ^50^ investigators could consider the option to elicit prospective participants' views on such issue, especially in research contexts when this has not previously been done or when employing innovative screening and diagnostic tools. When a positive decision is made on offering study results, some requirements should be met. Investigators should include a section in the informed consent form that solicits participants' preferences for whether or not they wish to receive individual results, and offers options regarding how any identified results will be returned. Investigators should also include a section where participants can identify a proxy to receive the results if they do not wish or are unable to receive them. Finally, investigators are urged to establish a direct, long‐lasting link with participants.

The present study is not without limitations. First, we cannot exclude possible selection bias, as participants were part of a previous study. However, this can be seen as an advantage as they may have already reflected upon the issue of the return of research findings. Second, participants might have answered the interviewer's questions in a manner that would be viewed favorably, introducing social desirability bias. To reduce this, the interviewer adopted techniques such as nominative questions and employed a nonjudgmental approach. Third, each interview was videotaped by a video‐maker. To reduce this contextual bias, the video‐maker was trained to limit intrusiveness and participants could choose where they would feel more at ease to be interviewed. Fourth, being dementia a sensitive topic to embark on, this may have had an effect on study participants in terms of difficult emotions and impaired openness. To mitigate this information bias, we established rapport with study participants by telephone before data collection, and fostered reciprocal trust through dialog. Finally, the language (ie, Italian) and nationality (ie, Swiss) of the participants may limit the generalization of our findings, which should be interpreted and applied cautiously to populations of other geographic and linguistic regions.

## 5 CONCLUSIONS

We found that, in the context of a dementia prevalence study, participants expect their rights both to know and not to know their results to be respected, provided the meaning and potential clinical implications of study findings have been previously assessed and clarified. Considering the implications of the issue of result disclosure for decision to participate and the representativeness of dementia epidemiological study samples, investigators should offer participants an ample set of options on the return of their individual‐specific results. Epidemiologists' primary roles are the design and conduct of scientific research and the public health application of scientific knowledge.^42^ This includes the reporting of results not only to the scientific community and society but also to research participants.^42^ However, it is not clear if this applies to aggregate or individual‐specific results. A trade‐off between anonymising data and being able to provide individual‐specific results may exist and should be adequately accounted for. Since formal guidance is lacking, we call for evidence‐based guidelines on how to assess the duty to return individual‐specific results in dementia epidemiological research. Finally, ethics committees should support the development of plans to return individual research results, and additionally assess whether they were developed in alignment with prospective participants' needs, preferences, and values.

## CONFLICT OF INTEREST

The authors declare no conflicts of interest.

## Supporting information


**Appendix** 1. Interview grid

## Data Availability

Due to the sensitivity of the information provided by participants, data are only available upon request.
